# QuickProbs 2: Towards rapid construction of high-quality alignments of large protein families

**DOI:** 10.1038/srep41553

**Published:** 2017-01-31

**Authors:** Adam Gudyś, Sebastian Deorowicz

**Affiliations:** 1 Institute of Informatics, Silesian University of Technology, Akademicka 16, 44-100 Gliwice, Poland

## Abstract

The ever-increasing size of sequence databases caused by the development of high throughput sequencing, poses to multiple alignment algorithms one of the greatest challenges yet. As we show, well-established techniques employed for increasing alignment quality, i.e., refinement and consistency, are ineffective when large protein families are investigated. We present QuickProbs 2, an algorithm for multiple sequence alignment. Based on probabilistic models, equipped with novel column-oriented refinement and selective consistency, it offers outstanding accuracy. When analysing hundreds of sequences, Quick-Probs 2 is noticeably better than ClustalΩ and MAFFT, the previous leaders for processing numerous protein families. In the case of smaller sets, for which consistency-based methods are the best performing, QuickProbs 2 is also superior to the competitors. Due to low computational requirements of selective consistency and utilization of massively parallel architectures, presented algorithm has similar execution times to ClustalΩ, and is orders of magnitude faster than full consistency approaches, like MSAProbs or PicXAA. All these make QuickProbs 2 an excellent tool for aligning families ranging from few, to hundreds of proteins.

Multiple sequence alignment (MSA) is of crucial importance in life sciences. The ability to reveal evolutionary and structural relationships between sequences makes MSA the basic tool in a number of biological analyses, including phylogeny, structure prediction, gene finding, and many others. Rapid dissemination of high throughput sequencing technologies causes sequence databases to grow exponentially^1^. To face this, the development of alignment algorithms able to process thousands of sequences in a reasonable time is required.

Among many proposed heuristics for finding multiple sequence alignments, progressive scheme has become the most popular. It consists of three steps: (I) estimating evolutionary distances between sequences, (II) building a guide tree based on the distances, (III) greedy alignment of sequences in the order described by the tree. The classic representative of progressive aligners with more than 50 thousand citations (Google Scholar, October 2016) is ClustalW^2^. The greatest disadvantage of progressive algorithms is the propagation of mistakes from bottom levels of the guide tree to the final result. A lot of techniques have been introduced to counter this issue. Historically, the first approach was to fix errors made at the progressive stage by iteratively refining the output alignment^3^. This idea has been successfully adopted by a number of algorithms like MAFFT^4^, MUSCLE^5^, or MSAProbs^6^. A different iteration scheme has been introduced in ClustalΩ^7^ which combines recalculations of a guide tree and profile hidden Markov models on the basis of a preliminary alignment. This results in a superior accuracy for large protein families. An alternative way of facilitating progressive heuristics is to prevent mistakes during alignment construction. This can be achieved in various ways. One method is to employ information from suboptimal alignments, e.g., by calculating posterior probabilities on the basis of pair hidden Markov models (ProbCons^8^), partition function (ProbAlign^9^), or both of those (MSAProbs). Another approach is incorporating knowledge from other pairwise alignments when processing given pairs of sequences/profiles. The technique is known as consistency, and has originally been used in T-Coffee^10^. Consistency has been proven to significantly elevate alignment quality and has been successfully adopted in different variants by a number of progressive (MAFFT^11^, ProbCons, MSAProbs), and non-progressive algorithms (PicXAA^12^). However, a substantial drawback of consistency-based methods, i.e., excessive computational complexity with respect to the number of sequences, limits their applicability to families of approximately hundred members. Consequently, algorithms allowing thousands or more sequences to be aligned like Kalign2^13^, Kalign-LCS^14^, FAMSA^15^, ClustalΩ, or MAFFT in PartTree mode^16^ do not use consistency.

In this article, we give a new insight into the effect of refinement and consistency on the progressive alignment. We investigated large sequence sets showing that accuracy of the aforementioned techniques scales unsatisfactorily with the number of sequences. In particular, when sets of hundreds or thousands of sequences are of interest, existing refinement variants have little effect on alignment quality, while consistency decreases it by introducing more noise than relevant information. We present new ideas to overcome those issues. i.e., column-oriented refinement and selective consistency.

The research was based on QuickProbs algorithm^17^ which is a successor of MSAProbs-one of the most accurate multiple sequence aligners^6^. Thanks to the utilization of massively parallel architectures, QuickProbs is order of magnitude faster than MSAProbs preserving quality of the results. We introduce QuickProbs 2, which is a significant improvement over its predecessor. Column-oriented refinement converges to alignments of higher quality than existing methods, while selective consistency incorporates most relevant information from pairwise alignments, effectively reducing the number of mistakes in a progressive scheme, also for large sets of sequences. Moreover, selectivity decreases dramatically computational effort related to consistency. This, together with optimized implementation, allows QuickProbs 2 to produce alignments superior to its forerunner in a fraction of the time. As a result, presented algorithm is the most accurate aligner when investigating protein families ranging from few to hundreds of sequences. Facilities like nucleotide mode or bulk processing further extend the usability of QuickProbs 2.

## Methods

In this paper, we introduce QuickProbs 2, a novel algorithm for multiple sequence alignment. It consists of four stages: (I) calculation of posterior probability matrices, (II) construction of the guide tree, (III) consistency transformation, (IV) construction of the final alignment followed by the iterative refinement. Posterior probability matrices are calculated for all sequence pairs on the basis of hidden Markov model^18^ and partition function^19^. The matrices are further employed to establish maximum expected accuracy alignments. Alignment scores are used to estimate pairwise distances which are given as an input for the weighted UPGMA algorithm^20^ for guide tree construction. In order to incorporate information from all pairwise alignments when aligning given pairs of sequences/profiles, posterior matrices are multiplied by each other during consistency transformation. Then, the sequences are progressively aligned in the guide-tree order with a use of the transformed posterior matrices. This is followed by the iterative refinement.

The most important advances with respect to existing methods were achieved at stages III and IV. QuickProbs 2 has been equipped with a novel column-oriented refinement and selective consistency, which are described further in following subsections. A separate subsection concerns other algorithmic improvements and new facilities introduced in the presented algorithm. Finally, we describe in detail benchmark datasets and measures used for quality assessment.

### Column-oriented refinement

Refinement was designed to overcome the most important disadvantage of progressive algorithms–misalignments caused by the propagation of errors from early progressive steps up the guide tree. Usually, the procedure employs an iterative scheme of alternate splits and realignments and incorporates an objective function for results evaluation. A number of refinement strategies were investigated in the literature^21^,^22^,^23^. Amongst them random and tree-guided approaches have become the most common in MSA algorithms.

First revision of QuickProbs, similarly to ProbCons or MSAProbs, employs the former idea: each refinement iteration splits an alignment randomly into two horizontal profiles and realigns them after removing columns containing only gaps. No objective function is incorporated. The substantial drawback of the procedure is that the larger the number of sequences, the smaller the chance of producing profiles with gap-only columns. As a result, no columns are removed in the majority of cases and the realigned profile is likely to be the same as the input one. Therefore, for numerous sets, consecutive random refinements give no improvement in accuracy. An alternative approach, incorporated e.g. by MUSCLE or MAFFT, is tree-guided refinement. It splits the alignment by breaking randomly selected branch in the guide tree. As gap-only columns are more likely to occur due to gathering phylogenetically related sequences in subprofiles, this approach can potentially be more successful when large protein families are investigated.

**Figure 1:**
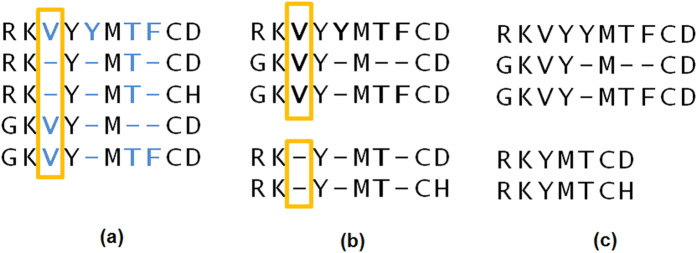
Iteration of column-oriented refinement: (**a**) one of the candidate columns (in blue) is randomly selected as a splitter (bounded with an orange box); (**b**) the alignment is divided into two profiles according to the presence of gaps in the selected column; (**c**) gap-only columns are removed which is followed by profile realignment.

We present a new approach to the refinement which considers columns containing at least one gap. The algorithm selects randomly one of those columns and splits the alignment into two profiles depending on the gap presence in this column. As a result, at each refinement iteration at least one profile is shortened increasing significantly the chance of rearranging the alignment and producing a higher quality outcome. This type of refinement will be referred to as *column-oriented* and, as experiments show, it is superior to the random and tree-guided approaches, especially for large sequence sets. Figure 1 presents the application of column-oriented refinement on example alignment.

An important observation is that the number of gaps *g* in a column, according to which an alignment is divided, affects the sizes of the resulting profiles. The closer is *g* to the half of the sequence set size *N*, the more balanced is the division. To investigate the effect of imbalance in profile splitting on alignment quality columns were sorted with respect to 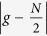. Then, an assumed percentage from the beginning or from the end was considered in the random selection (these correspond to the bias towards respectively, more or less balanced splits).

The refinement is often facilitated by introducing an objective function. The usage of *unsupervised* SP score under assumed substitution matrix and gap penalty model (not to confuse with *supervised* SP score calculated on the basis of the reference alignment) is amongst the most popular^5^,^11^. Nevertheless, maximizing unsupervised SP score does not necessarily converge to biologically meaningful alignments^22^,^24^, particularly for consistency algorithms^22^. In the research, we suggest alignment length to be used as a straightforward and effective measure for refinement supervision. Intuitively, misalignments at consecutive progressive steps accumulate, causing blocks of conserved symbols to be shifted with respect to each other. As a result, one can expect erroneous alignments to be longer than those correctly identifying evolution of sequences. This hypothesis is supported by the observation that average alignment length declines as quality increases in consecutive refinement iterations. Therefore, we introduced to the refinement an acceptance criterion of non-increasing alignment length which further improved the convergence.

In the research, we also examined entropy-based acceptance rule of non-decreasing *trident score* (see ref. 25 for the definition). The method employs three components for column scoring: amino-acid conservation, stereochemical properties, and the presence of gaps. The reasoning behind was that properly aligned columns which are structurally and functionally conserved should be characterized by lower entropy.

An open issue is the susceptibility of presented refinement approaches to the over-alignment, i.e. the generation of too short alignments. Most of the benchmark datasets, including those used in our study, are based on structure, not phylogeny. As a result, tight alignments are preferred, despite being improper from the evolutionary point of view^26^. This problem is common for the majority of sequence alignment methods and few attempts have been made to counter this issue with PRANK^26^,^27^ and MAFFT^28^ being the examples.

### Selective consistency

As opposed to refinement, the consistency aims at preventing misalignments in the progressive scheme rather than eliminating them afterwards. To reduce the chance of making errors, the consistency employs information from all pairwise alignments when aligning a pair of two particular sequences. Even though this approach has been successfully applied in a number of progressive MSA algorithms, the excessive computational cost limits its applicability to sets of hundreds of sequences. Sievers *et al*.^29^ made an exhaustive study on scalability of MSA algorithms examining the effect of addition of homologous sequences to the reference set on the alignment accuracy. For all observed methods quality deteriorated when more than 50 sequences were added. The decay was especially steep for several consistency-based methods (e.g., MSAProbs, ProbCons) suggesting that for larger sets of sequences, noise exceeds relevant information. This, however, has not been explicitly verified. The recent experiments on applying consistency (particularly, MAFFT G-INS-1 algorithm) on larger sets of sequences required days of computations and prohibitive amount of RAM^30^. It is also unclear whether promising quality of the results was due to performing full pairwise alignments for guide tree construction or thanks to the consistency.

In QuickProbs 2, similarly to its predecessor, consistency relies on transforming posterior probability matrices calculated at stage I. Let *U* indicate a set of input sequences and *S*_*xy*_ be a posterior matrix for sequences *x, y* ∈ *U*. In QuickProbs 1, the consistency incorporates to *S*_*xy*_ information from all other sequences according to the formula


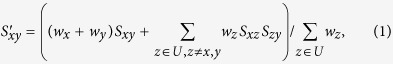


with *w*_*u*_ being the weight of sequence *u* established during tree construction. The inclusion of the information from *z*, i.e., the addition of *w*_*z*_*S*_*xz*_*S*_*zy*_ component will be referred to as a *relaxation* of *S*_*xy*_ over *z*. A single consistency transformation relies on relaxing all posterior matrices through all sequences. This process can be iterated, i.e., the matrices calculated as an output of one transformation can be used as an input for another.

The time complexity of *O*(*N*^3^*L*^3^), with *L* being the sequence length, makes this stage very time consuming. Precisely, posterior matrices are represented in a sparse form with a sparsity coefficient *β* < 1. As presented in Supporting information to ref. 17, the time complexity depends on the structure of sparse matrices and varies from *O*(*β*^2^*N*^3^*L*^3^) to *O*(*βN*^3^*L*^3^). Nevertheless, as QuickProbs comes with a fast relaxation algorithm suited for graphics processors, we were able to investigate the effect of consistency on sets exceeding one thousand sequences. As presented in the experimental section, the procedure decreased alignment quality for protein families of such sizes.

The challenge which naturally arises, is to apply consistency only on sequences carrying most of the information. Particularly, we examined whether there is a correlation between information content and evolutionary relationship of sequences involved in the consistency. For this purpose we introduce *selective consistency*. Given *x, y, z* sequences and *d*_*xz*_, *d*_*yz*_ distances, posterior matrix *S*_*xy*_ is relaxed over sequence *z* if an *aggregation function α*(*d*_*xz*_, *d*_*yz*_) fulfills given condition. In the research we investigated two different *d*_*xy*_ measures:

- score-based distance calculated at stage I, ranked and normalized to [0, 1] interval,
- tree-guided distance defined as a number of nodes in a minimal subtree containing both *x* and *y*.

Maximum, minimum, or sum can be used as examples of aggregation function *α*. Selectivity was applied either by:

- deterministically thresholding *α* on arbitrary value *T*,
- applying stochastic filtering.

The latter requires defining a *filter function F* which maps the value of the aggregation function *α*(*d*_*xz*_, *d*_*yz*_) to the probability of performing relaxation over sequence *z*, i.e.: *F*(*α*): *α*(*d*_*xz*_, *d*_*yz*_) → [0, 1]. Shape of the filter function determines which sequences are preferred in the consistency procedure (e.g. closely or distantly related). The procedure of stochastic selectivity for matrix *S*_*xy*_ over sequence *z* works according to the following steps:

1. calculate the value of *α*_0_ = *α*(*d*_*xz*_, *d*_*yz*_) according to the assumed distance measure *d* and aggregation function *α*,
2. determine the value of the filter function *F*(*α*_0_),
3. sample a random number *p* from the uniform distribution [0, 1],
4. if *F*(*α*_0_) ≤ *p*, perform relaxation of *S*_*xy*_ over *z*.

**Figure 2:**
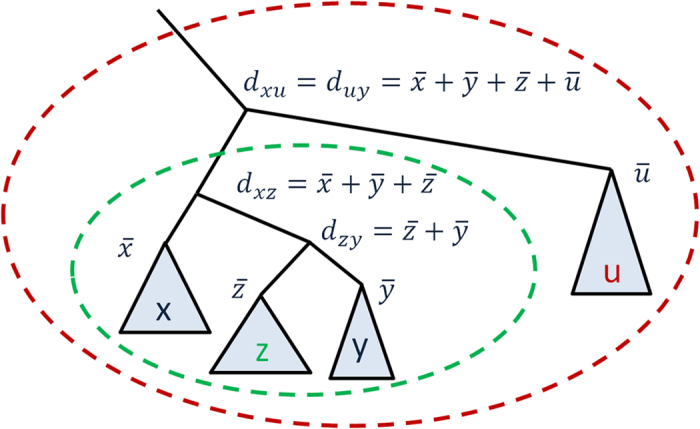
Tree-guided selective consistency of *S*_*xy*_ posterior matrix with threshold *T*. Triangles represent subtrees with sequences *x, y, z*, and *u*. At each node, a size of a subtree is given. In the example, selectivity procedure accepts relaxation of *S*_*xy*_ through *z* as *α*(*d*_*xz*_, *d*_*zy*_) ≤ *T* (green oval). At the same time it excludes sequence *u* from the consistency due to *α*(*d*_*xu*_, *d*_*uy*_) > *T* (red oval).

As combination of tree-based distances and deterministic thresholding rendered superior results, we explain this variant of selectivity in Fig. 2.

The side effect of selectivity is the variability in the number of relaxations performed for different posterior matrices. Consequently, the larger the number of sequences undergoing consistency transformation, the weaker is the information from the original *S*_*xy*_ compared to the matrices it is multiplied by. E.g., a matrix relaxed by thirty sequences contributes to the transformation ten times less than that relaxed by three. To overcome this, we additionally analysed the effect of multiplication of *S*_*xy*_ elements by a coefficient *h*_*xy*_. The value of *h*_*xy*_ is set individually for each matrix and varies linearly from 1, when no relaxations of *S*_*xy*_ are performed, to user-defined value *h*, when maximum number of relaxations under chosen selectivity settings is done (200 in our case). This allows sets of different sizes to be handled properly.

### Other algorithmic improvements

In spite of focusing QuickProbs 2 research on extending refinement and consistency stages, calculation of posterior matrices was also a subject to some modifications. Quality improvements include replacing Gonnet160 matrix for partition function calculation by VTML200, which was proven to be more accurate^31^. This was followed by training partition function parameters, i.e., gap penalties and temperature on BAliBASE 3^32^ benchmark with a use of NOMAD algorithm^33^ for optimization of non-smooth functions. Another changes were introduced in order to shorten execution time. They include redesigning graphics processor calculations to handle sequences of any length, optimization of both CPU and GPU codes, and using more efficient memory allocation. As a result, posterior calculation stage in QuickProbs 2 is more accurate than its predecessor, being noticeably faster. The typical speedup of stage I on moderately-sized families was twofold. When families of long proteins were investigated like BB20010 from BAliBASE^32^ (29 sequences with 1,045 amino acids on average) the computation of posterior matrices was 10 times faster than in QuickProbs 1.

The presented algorithm is equipped with a nucleotide mode in which HOXD substitution matrix^34^ and GTR evolutionary model^35^ are used. Accurate mode, which in QuickProbs 1 adjusted a sparsity coefficient in posterior matrices, is no longer supported due to excessive computation time and lack of significant impact on the results.

Due to different behaviour of consistency depending on the set size, the number of transformations is adjusted to the number of sequences (2 for *N* < 50, 1 otherwise). It was also discovered that for two consistency transformations, 30 iterations of refinement instead of default 200 is sufficient to get satisfactory convergence.

As QuickProbs 2 employs OpenCL, it can be executed on different massively parallel devices like NVidia and AMD GPUs. Moreover, presented software has also the ability to be run on central processor without OpenCL. For convenience, QuickProbs 2 is equipped with a *bulk mode* allowing any number of sequence sets to be processed during a single run. Necessity of storing posterior matrices for all pairs of sequences causes memory to be the major limiting factor for the set size. For this reason, QuickProbs 2 gives the opportunity to fit analysis in a user-specified amount of RAM by decreasing the sparsity coefficient in posterior matrices. Naturally, the adjustment affects the quality and is possible only within certain boundaries.

### Accuracy assessment

Accuracy of algorithms was assessed on several benchmark datasets that come with reference alignments. Those were BAliBASE^32^, PREFAB^5^, SABmark^36^, HomFam^37^, and BaliFam^29^. The three former were downloaded in a standardized FASTA format from Robert Edgar’s Webpage^38^ and consist of small and moderate sequence sets (up to tens of sequences in the majority of cases). The latter were constructed by enriching respectively, Homstrad^39^ and BAliBASE benchmarks, with full protein families from Pfam^40^. Number of sequences in BaliFam sets is in the order of 1,000 while Homfam contains much larger families of more than 100,000 members. Both benchmarks were postprocessed by removing duplicated sequences which appear numerously due to generation protocol. This was motivated by the fact that duplicates may affect the accuracy of analysed algorithms and can be straightforwardly restored after alignment has finished.

Postprocessed BaliFam contained 218 sets with 934 sequences on average. As the major part the research focuses on the scalability of presented methods with respect to the number of sequences, BaliFam was recursively resampled to obtain less numerous sets: initial benchmark into two sets of 800 sequences, each of those into two sets of 600, and so on. Finally, elements at the same level of the pyramid were gathered forming sets referred to as BaliFam-800 × 2, BaliFam-600 × 4, BaliFam-400 × 8, and BaliFam-200 × 16. This protocol includes smaller sets in the larger ones and preserves representativity for all problem sizes. As for the HomFam, after duplicate removal, all its sets were randomly downsampled to 1,300 members with a guarantee of preserving sequences present in the reference alignments. This was motivated by the fact that original HomFam sets were too large to be processed by QuickProbs 2 due to memory requirements. Sampled benchmark will be referred to as HomFam^1K^ and contained 94 families with 1,093 sequences on average. Detailed histograms of family sizes in BaliFam and HomFam^1K^ are presented in Supplementary Figure 1.

Quality evaluation was performed with well-established metrics related to reference alignments. Those are supervised sum of pairs (SP) and total column (TC) scores defined as a fraction of correctly aligned symbol pairs and columns, respectively. When a single quality measure was needed, e.g., for visualization, a geometric mean 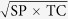 of the aforementioned scores was employed. Separate charts for SP and TC measures are given as Supplementary Figures.

## Results

### Refinement

**Figure 3:**
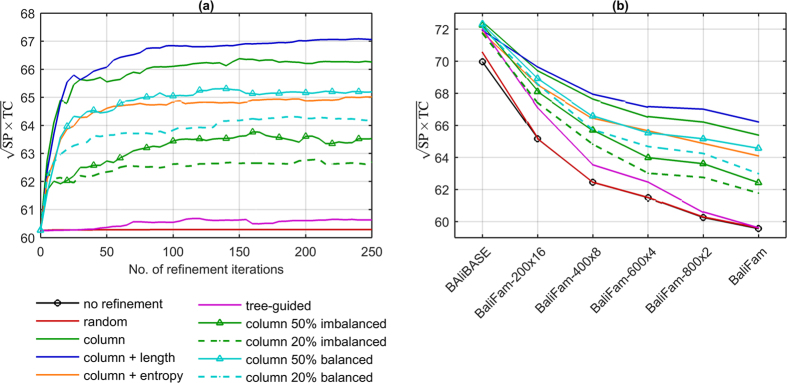
Comparison of refinement strategies: (**a**) effect of consecutive iterations on BaliFam-800 × 2, (**b**) scalability with respect to the number of sequences in a set after 200 refinements.

In the initial experiments, we investigated random and tree-guided refinements together with different variants of novel column-oriented procedure. As refinement was acquired by alignment algorithms formerly to consistency, the latter was disabled in this experimental part. BaliFam-800 × 2 benchmark was selected as a representative of large protein families instead of BaliFam because it contains twice as many sets which reduces results variability. The effect of consecutive refinement iterations is presented in Fig. 3a, while scalability of refinement with respect to the set size after 200 iterations can be observed in Fig. 3b.

As charts show, for numerous protein families such as those in BaliFam-800 × 2, consecutive random refinements gave no gain in the accuracy. Moreover, random procedure was profitable only for BAliBASE and starting from BaliFam-200 × 16 it had no effect on the results. The performance of tree-guided refinement was noticeably better, however the improvement declined with the increasing number of sequences. The opposite situation occured in the case of column-oriented refinement. Not only it was superior to the competing approaches, but the advance over non-refined output was characterized by perfect scalability. Namely, it increased from 2% on BAliBASE to almost 7% on BaliFam, confirming the selection of gap-only columns to be the choice for large protein families. When analysing the effect of split imbalance on alignment quality, it is visible that narrowing the subset of columns considered in the selection to 50% or 20% of most balanced/imbalanced positions caused accuracy decay. Consequently, the version without preference was chosen for further investigation.

The final refinement experiments concerned the effect of different acceptance rules. Those were non-increasing alignment length and non-decreasing entropy score. As presented in Fig. 3, the former improved refinement convergence for larger sequence sets being only slightly inferior to unsupervised variant on BAliBASE. In contrast, entropy scoring performed unsatisfactorily on all analysed sets. As a result, column-oriented refinement with length supervision was selected for QuickProbs 2. Charts presenting influence of refinement on SP and TC measures separately can be found in Supplementary Figure 3.

**Figure 4:**
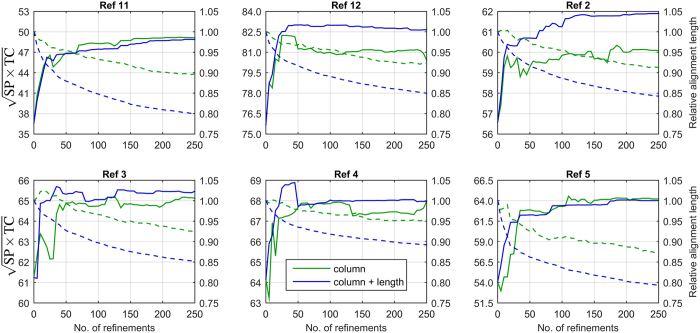
Comparison of unsupervised and supervised column-oriented refinement on BaliFam reference sets. Alignment qualities and their relative lengths are represented as continuous and dashed lines, respectively.

To investigate the accuracy of column-oriented refinement in different classes of alignment problems, all BaliFam subsets were analysed independently. They correspond to BAliBASE 3 reference sets representing: equidistant sequences of 0–20% (ref. 11) and 20–40% (ref. 12) identity, families with orphans (ref. 2), divergent subfamilies (ref. 3), large extensions (ref. 4), and large insertions (ref. 5). Figure 4 presents quality results together with relative alignment lengths for the unsupervised and supervised algorithms. The first observation concerns the relative alignment lengths decreasing with consecutive refinement iterations for both algorithm variants. The reduction rate was larger in the case of the supervised procedure. When comparing accuracies, the largest advance of the supervised variant was observed on refs 2,12. In the case of refs 3,4 the advantage was less evident. On refs 5,11 both refinement variants performed similarly. An interesting observation is that alignments produced by the supervised variant were significantly shorter than equally accurate results of the unsupervised procedure. One of possible explanations is that introducing the acceptance criterion of non-increasing length causes output alignments to be tighter in gapped regions which are outside benchmark evaluation blocks. To investigate the reconstruction quality of those regions, synthetic, phylogeny-aware families like those suggested by^28^ should be employed. Therefore, the susceptibility of presented approach to over-alignment is an open issue.

### Consistency

**Figure 5:**
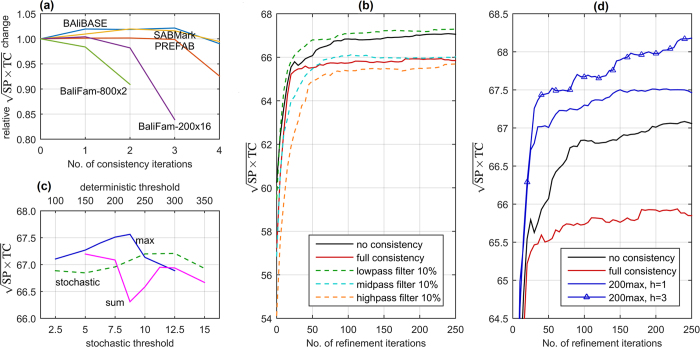
(**a**) Effect of consistency iterations on selected benchmarks. Analysis of consistency on BaliFam-800 × 2: (**b**) effect of stochastic distance-related filtering, (**c**) selectivity variants for closely related sequences, (**d**) weighting original posterior matrices by *h*_*xy*_ ∈ [1, *h*] coefficient. Alignment qualities from charts (**a**,**c**) measured after 200 refinements.

**Figure 6:**
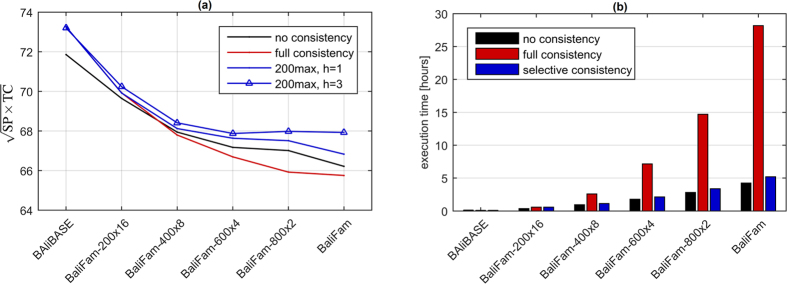
Scalability of consistency after 200 refinement iterations: (**a**) alignment quality, (**b**) execution time on *desktop* configuration with GeForce GPU. Since each benchmark on the horizontal axis contain twice less test cases then its predecessor, times were multiplied as if all sets were equally numerous.

Figure 5a shows the effect of the traditional (non-selective) consistency iterations on selected benchmarks after 200 refinements. For smaller sets (BAliBASE, PREFAB, SABmark) the consistency introduced relevant information, elevating result quality. Nevertheless, at the same time it interposed noise which accumulated for large sets of sequences causing accuracy decay even after the first iteration (800 × 2). Figure 5b proves the consistency to be harmful on large benchmarks independently of refinement iteration. Figure 6a shows that the noise started to exceed relevant information for N > 400.

Clearly, selecting only part of sequences for consistency can potentially increase the effectiveness of the procedure. To investigate a correlation between information content and evolutionary relationship of sequences involved, we applied triangle stochastic filters with an expected acceptance rate of 10%. Those were low-pass, mid-pass and high-pass filters which promoted consistency over respectively: closely, mildly, and distantly related sequences. The shapes of the filter functions are presented in Supplementary Figure 2. Distances were calculated as alignment scores from stage I, ranked and normalized to [0, 1], the sum was used as an aggregation function *α*. Figure 5b shows, that closely related sequences introduce more information to the consistency, thus should be preferred in the selection. Besides stochastic filtering, deterministic selectivity based on a structure of the guide tree was examined. The consistency over sequence *z* was performed when sum or maximum of tree-based distances *d*_*xz*_ and *d*_*yz*_ was smaller than assumed threshold *T*. The comparison of selectivity strategies (Fig. 5c) demonstrates the deterministic variant with maximum function thresholded at 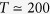 to perform the best. It was superior to the version without consistency independently of refinement iteration with an exception of *r* = 0 point where no-consistency won (Fig. 5d). The effect of consistency being profitable only when paired with refinement was not observed on smaller sets. To gain deeper insight into this phenomenon, more detailed investigation on interdependencies between consistency and refinement is required.

As a next step, we analysed the effect of amplification of the original *S*_*xy*_ signal by multiplying its elements by coefficient *h*_*xy*_ linearly scaled in [1, *h*] interval. The largest improvement in the alignment quality was for *h* = 3 (see Fig. 5d) and was observed for all sets of 200 or more sequences (Fig. 6a). At the same time, automatic adjustment of *h*_*xy*_ depending on the number of relaxations, prevented from accuracy drop on BAliBASE which contains much smaller sets. Charts presenting influence of consistency on SP and TC measures separately can be found in Supplementary Figures 4 and 5.

The crucial feature of selective consistency is its low computational requirements. For N ≥ 200 an approximate number of relaxations for each posterior matrix is constant. As a result, time complexity of the procedure is *O*(*N*^2^*L*^3^) which is a noticeable improvement over full consistency variant. The comparison of execution times (Fig. 6b) shows that for large sets of sequences, time overhead related to selective consistency was negligible compared to other QuickProbs 2 stages.

### Comparison with other algorithms

The comparison of alignment software on benchmark datasets is given in Table 1. The algorithms were executed on *desktop* configuration (details of hardware configurations will be explained later). Software packages suited for parallel processing were run with 12 processing threads to fully utilize multi-core architecture of the CPU.

**Figure 7:**
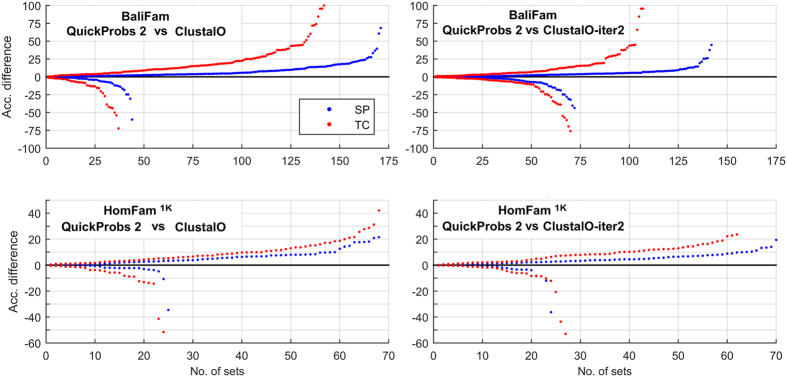
Detailed comparison of QuickProbs 2 and ClustalΩ variants on BaliFam and HomFam^1K^ benchmarks. For each quality measure (SP/TC) differences on individual protein families were sorted and plotted as two independent series. The points above the horizontal axis represent sets on which QuickProbs 2 was superior, the ones below correspond to the opposite situation.

For small sets of sequences (BAliBASE, PREFAB, and SABmark) QuickProbs 2 competes with other consistency-based algorithms. Experiments show QuickProbs 2 to overcome them by a small margin (the distance to the second best does not exceed one percentage point on both SP and TC) with an exception of SABmark where GLProbs^41^ took the lead. This can be explained by GLProbs being equipped in local alignment Markov models, which are especially profitable on distantly related sequences in SABmark. PicXAA, the only non-progressive algorithm in the comparison, is also inferior to QuickProbs 2. For large sets of sequences (BaliFam and HomFam^1K^), consistency methods became inapplicable due to hardware limitations. Moreover, the accuracy of consistency was often unsatisfactory, as in the MSAProbs case. For these reasons, ClustalΩ became the choice when numerous alignments were of interest. Nevertheless, thanks to the column-oriented refinement and selective consistency, QuickProbs 2 was noticeably more accurate than ClustalΩ on both large sets. E.g., the greatest advantage observed on BaliFam in TC score corresponds to almost 25% more successfully aligned columns. When one considers ClustalΩ with two combined iterations enabled, QuickProbs 2 was still superior by a fair margin. Figure 7 presents a detailed comparison of the presented algorithm and ClustalΩ variants on BaliFam and HomFam^1K^ benchmarks. For all families in a benchmark, absolute advantages of QuickProbs over competing software in SP and TC measures were determined. For each measure, the differences were sorted and plotted on a chart as two independent series. The points above the horizontal axis represent sets on which QuickProbs 2 was superior, the ones below correspond to the opposite situation. This way one can asses on what portion of the dataset and to what extent one algorithm performed better than the other. The advance of QuickProbs 2 over default variant of ClustalΩ is clear: on both analysed benchmarks our algorithm was superior to the competitor on approximately 3/4 families. This was also the case for ClustalΩ-iter2 on HomFam^1K^. A bit different situation was for BaliFam, where enabling combined iterations noticeably improved ClustalΩ results. Though, it was still clearly inferior to QuickProbs 2.

**Figure 8:**
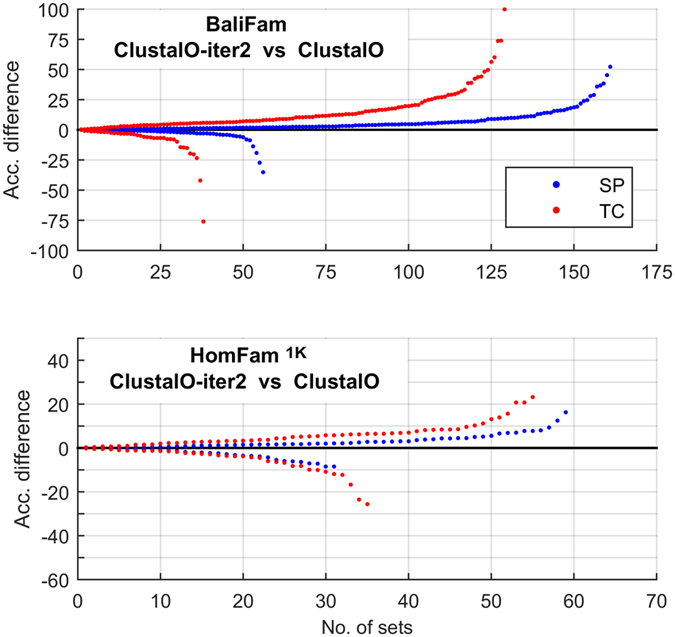
Detailed comparison of ClustalΩ-iter2 over ClustalΩ on BaliFam and HomFam^1K^ benchmarks. For each quality measure (SP/TC) differences on individual protein families were sorted and plotted as two independent series. The points above the horizontal axis represent sets on which ClustalΩ-iter2 was superior, the ones below correspond to the opposite situation.

The effect of presented algorithm being worse than ClustalΩ on several test cases is natural and is visible also when comparing other algorithms. For instance, combined iterations were reported to significantly elevate the quality of ClustalΩ^7^,^29^ results. However, when analysing differences on particular protein families, there are sets for which default configuration is more accurate (Fig. 8). This can be explained by the high diversity of alignment problems, which hinders the development of algorithms superior to the competitors systematically on all test cases. Therefore, the statistical analysis of the results is necessary to properly assess performance of investigated methods. Significance of reported differences was verified with a use of Wilcoxon signed-rank test (Table 2). To control family-wise error at *α*=0.05, the Bonferroni-Holm correction was applied. Low *p*-values for BaliFam and HomFam^1K^ give strong evidence that QuickProbs 2 is currently the best algorithm for alignment of large sets of sequences also when compared to ClustalΩ-iter2 and MAFFT-L-INS-i. The lack of significance was observed in few cases concerning small sets only (including the advantage of GLProbs over QuickProbs 2).

Superior accuracy of QuickProbs 2 on large protein families coincides with reasonable computational requirements. QuickProbs 2 is comparable to default mode of ClustalΩ in terms of execution times and orders of magnitude faster than consistency-based methods (MSAProbs needed over a week to complete BaliFam, QuickProbs 1 failed to run properly due to memory requirements). As QuickProbs 2 employs OpenCL, it can be executed on different massively parallel devices like NVidia and AMD GPUs. Moreover, presented software has also the ability to be run on central processor without OpenCL. As experiments on different hardware platforms show (Table 3), CPU variant is 3–10 times slower than GPU version, though still faster than other algorithms based on consistency.

The bottleneck of QuickProbs 2 are the memory requirements, particularly the neccessity to store posterior matrices for all pairs of sequences. E.g. 8 GB of RAM was needed to process 1,000 sequences of length 100 or 300 sequences of length 500. When 64 GB was available, presented algorithm successfully aligned familes of 1,300 proteins with 500 amino acids.

## Discussion

Constantly growing availability of genomic and proteomic data opens new opportunities in life sciences. Yet, it is a major challenge facing algorithms for sequence analyses, including multiple sequence alignment. Increasing number of sequences is one of the most important factors determining the difficulty of the MSA problem. In our research we have confirmed refinement and consistency, two most popular quality-aimed techniques employed by progressive aligners, to be ineffective or even harmful for sets of hundreds and more sequences. We present QuickProbs 2, a multiple alignment algorithm equipped with novel column-oriented refinement and selective consistency. It scales well with the number of sequences offering significantly better accuracy than ClustalΩ—the previous leader for analysing large sets of sequences. For less numerous sets (*N* < 100), when methods based on full consistency like MAFFT-L-INS-i, MSAProbs, or PicXAA are applicable, QuickProbs 2 is still superior to the competitors. What is important, outstanding accuracy is obtained in a short time thanks to the utilization of massively parallel architectures.

By successfully extending applicability of refinement and consistency to approximately thousand of sequences, we showed that sets of different sizes require various treatment. An open issue though, is the scalability of presented ideas for families of tens or hundreds thousands of sequences that are common in Pfam database. This is caused by the memory requirements of QuickProbs 2, the main issue to be resolved in future releases. For such large sets of sequences ClustalΩ or MAFFT are still the choice.

Other factors contributing to the complexity of multiple alignment problem are sequence lengths, their evolutionary relationship, presence of long terminal fragments, etc. We believe that future development of MSA domain is impossible without better understanding of the influence of all these elements on alignment algorithms. Especially, in the light of recent, though questionable, discoveries concerning performance of chained guide trees in alignment of large sets of sequences^15^,^42^,^43^,^44^. Our research also leads to some observations that remain to be explained, e.g., the effect of consistency being profitable for large protein families only when paired with refinement. Deeper involvement of biological community, which by definition is the major recipient of multiple alignment algorithms, would considerately facilitate advances in this area of computational biology.

QuickProbs 2 executables together with source code are available at https://github.com/refresh-bio/QuickProbs. All examined datasets can be downloaded from http://dx.doi.org/10.7910/DVN/7Z2I4X. Web service for remote analyses is under development.

## Additional Information

**How to cite this article**: Gudyś, A. and Deorowicz, S. QuickProbs 2: Towards rapid construction of high-quality alignments of large protein families. *Sci. Rep.* **7**, 41553; doi: 10.1038/srep41553 (2017).

**Publisher's note:** Springer Nature remains neutral with regard to jurisdictional claims in published maps and institutional affiliations.

## Supplementary Material

Supplementary Information

## Figures and Tables

**Table 1:** Results for benchmark datasets on *desktop* configuration.

Algorithm	BAliBASE			PREFAB		SABmark			BaliFam			HomFam^1K^		
	time	SP	TC	time	SP/TC	time	SP	TC	time	SP	TC	time	SP	TC
QuickProbs 2	2:01	**88.1**	**61.8**	8:18	**74.2**	10	61.1	40.7	3:35:46	**84.7**	**54.8**	1:43:36	**87.7**	**72.0**
QuickProbs-acc	25:45	87.9	60.8	57:25	74.0	53	60.3	40.1	—	—	—	—	—	—
QuickProbs	5:17	87.8	60.7	15:37	73.6	20	60.3	40.1	—	—	—	—	—	—
MSAProbs	25:12	87.8	60.8	1:42:51	73.7	30	60.2	40.0	>8 days	60.9	34.5	>2 days	77.5	60.9
PicXAA-PF	3:20:51	87.8	59.3	13:31:09	71.2	3:35	59.0	38.4	—	—	—	—	—	—
PicXAA-HMM	2:13:35	86.5	56.4	9:12:24	71.1	2:50	59.3	39.0	—	—	—	—	—	—
GLProbs	40:12	87.9	59.3	2:06:36	72.4	58	**61.4**	**41.4**	—	—	—	—	—	—
MAFFT -L-INS-i	15:36	86.8	58.5	17:15	72.1	44	57.1	36.8	>3 days	77.0	43.4	23:26:06	85.2	67.9
ClustalΩ-iter2	1:07:32	84.8	56.7	2:35:46	71.0	2:52	55.2	35.7	11:11:17	83.7	51.8	3:23:56	85.1	68.6
ClustalΩ	4:56	84.2	55.9	14:19	70.0	18	55.0	35.5	1:27:21	79.9	44.5	46:10	84.1	67.2
MUSCLE	8:47	81.9	47.8	22:32	67.7	32	54.5	33.5	>4 days	52.1	22.3	31:42:23	70.6	50.8
Kalign-LCS	21	83.0	50.4	1:30	65.9	2	55.6	35.6	6:36	67.5	31.5	4:14	81.5	62.1
MAFTT -FFT-NS-2	1:38	81.7	47.5	8:03	68.0	54	53.2	33.0	13:13	66.0	28.8	3:50	79.1	58.1
Kalign2	26	81.1	47.1	1:39	65.5	2	52.4	32.6	11:25	66.6	31.0	5:43	77.4	57.5

All versions of QuickProbs were run on Radeon 7970 due to incompatibility of QuickProbs 1 with GeForce 980. Best quality results typed in bold. Execution times given in *hh*:*mm*:*ss* format. Line separates consistency (top) from non-consistency (bottom) methods.

**Table 2:** Statistical significance of the results.

Benchmark	QuickProbs-acc	MSAProbs	GLProbs	ClustalΩ	ClustalΩ-iter2	MAFFT-L-INS-i
BAliBASE	(0.02011)	(0.03038)	0.00006	<10^−15^	<10^−10^	<10^−9^
PREFAB	(0.01694)	<10^−6^	<10^−20^	<10^−20^	<10^−20^	<10^−20^
SABmark	0.00998	0.00685	(−0.05203)	<10^−14^	<10^−12^	<10^−12^
BaliFam	—	<10^−20^	—	<10^−13^	0.00100	<10^−10^
HomFam^1K^	—	<10^−11^	—	<10^−5^	<10^−5^	0.04713

*P*-values of TC differences between QuickProbs 2 and selected methods were measured with a use of Wilcoxon signed-rank test. Insignificant results at *α* = 0.05 are given in parentheses. The Bonferroni-Holm correction for multiple testing was applied independently on groups of benchamrks with small (BAliBASE, PREFAB, SABmark) and large (BaliFam, HomFam^1K^) protein families; minus sign indicates the advantage of the competing software.

**Table 3:** Execution times of QuickProbs 2 in GPU and CPU modes.

	*desktop*			*laptop*		*workstation*	
	i7 4930 K (6 × 3.4 GHz)			i7-4700MQ (4 × 2.4 GHz)		2 × Xeon E5-2670v3 (24 × 2.3 GHz)	
	64 GB RAM			8 GB RAM		128 GB RAM	
	Radeon 7970^1^ + GeForce 980^2^			Radeon 8970 M^3^		Quadro M6000^4^	
	Radeon	GeForce	CPU	GPU	CPU	GPU	CPU
BAliBASE	2:01	2:43	15:18	2:55	23:04	2:19	6:41
PREFAB	8:18	12:32	1:05:12	13:03	1:41:16	9:15	26:43
SABmark	10	18	27	11	35	18	20
BaliFam	3:35:46	5:15:40	34:25:29	—	—	5:13:22	14:00:03
HomFam^1K^	1:43:36	2:29:31	22:21:42	—	—	2:29:05	6:32:54

BaliFam and HomFam^1K^ were not analysed on *laptop* due to memory requirements. BAliBase, PREFAB, and SABmark were processed in bulk mode.

^1^2048 × 1.0 GHz, 3 GB RAM (228 GB/s), 15.30 driver, Windows 7 × 64.

^2^2048 × 1.2 GHz, 4 GB RAM (224 GB/s), 359.06 driver, Windows 10 × 64.

^3^1280 × 0.9 GHz, 4 GB RAM (154 GB/s), 15.11 driver, Windows 7 × 64.

^4^3072 × 1.0 GHz 12 GB RAM (317 GB/s), 352.55 driver, CentOS 7.1 × 64.
