# Supplementary material for: QuickProbs 2: Towards rapid construction of high-quality alignments of large protein families
Source: Sci Rep. 2017 Jan 31;7:41553. doi: 10.1038/srep41553 (PMC5282490; doi:10.1038/srep41553)
Supplement: Supplementary Information [file srep41553-s1.pdf]

QuickProbs 2: Towards rapid construction of high-quality alignments of large protein families–supplement

Adam Gudyś, Sebastian Deorowicz

August 28, 2016

Institute of Informatics, Silesian University of Technology, Akademicka 16, 44-100 Gliwice, Poland

Corresponding Author: Adam Gudyś, adam.gudys@polsl.pl

Algorithm parameters

Alignment software was executed with following parameters (*platform* and *device* are identifiers of OpenCL platform and device selected for computation).

- ClustalΩ (1.2.0): -i *input* -o *output* --threads=12
- ClustalΩ-iter2 (1.2.0): -i *input* -o *output* --threads=12 --iter=2
- GLProbs (1.0): *input* -o *output*
- Kalign2 (2.04): -quiet -i *input* -o *output*
- Kalign-LCS (2.04): -quiet -b upgma -d lcs_indel -i *input* -o *output*
- MAFFT-auto (7.221): --quiet --thread 12 --auto *input*
- MAFFT (7.221): --quiet --thread 12 *input*
- MSAProbs (0.9.7): *input* -o *output*
- MUSCLE (3.8.31): -quiet -in *input* -out *output*
- PicXAA-PF (1.0): -PF *input*
- PicXAA-HMM (1.0): -PHMM *input*
- QuickProbs (1.01): -p *platform* -d *device* *input* -o *output*
- QuickProbs-acc (1.01): -p *platform* -d *device* *input* -o *output* -q
- QuickProbs 2 (2.04): -p *platform* -d *device* *input* -o *output*

Supplementary Figures

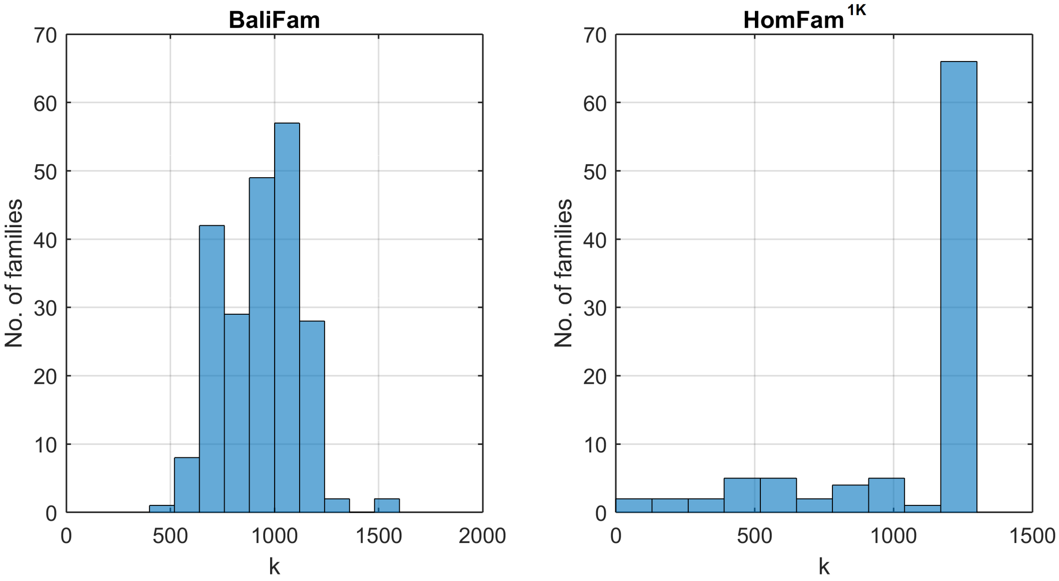

Supplementary Figure 1: Histograms of protein families sizes in BaliFam and HomFam^{1K} benchmarks.

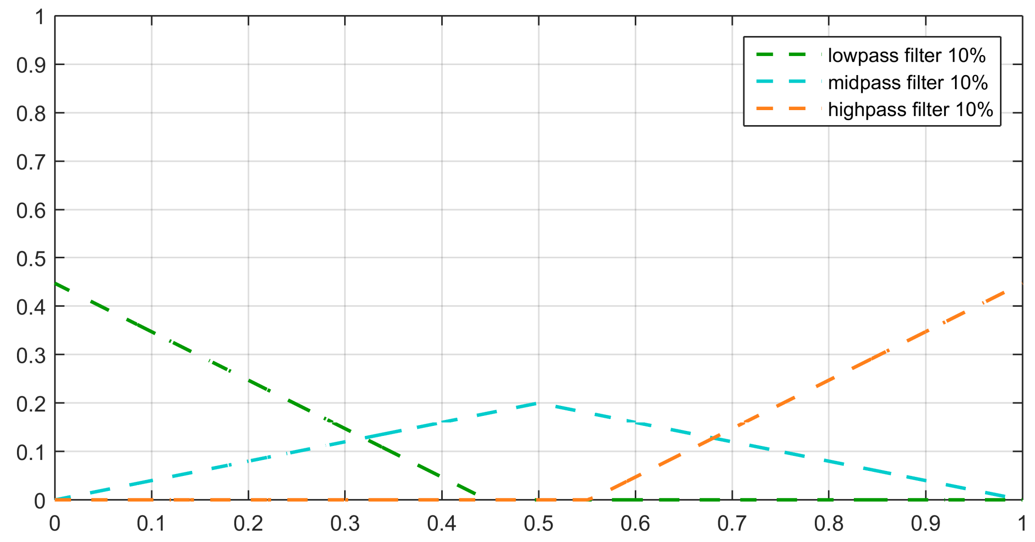

Supplementary Figure 2: Shapes of stochastic filters used in selective consistency.

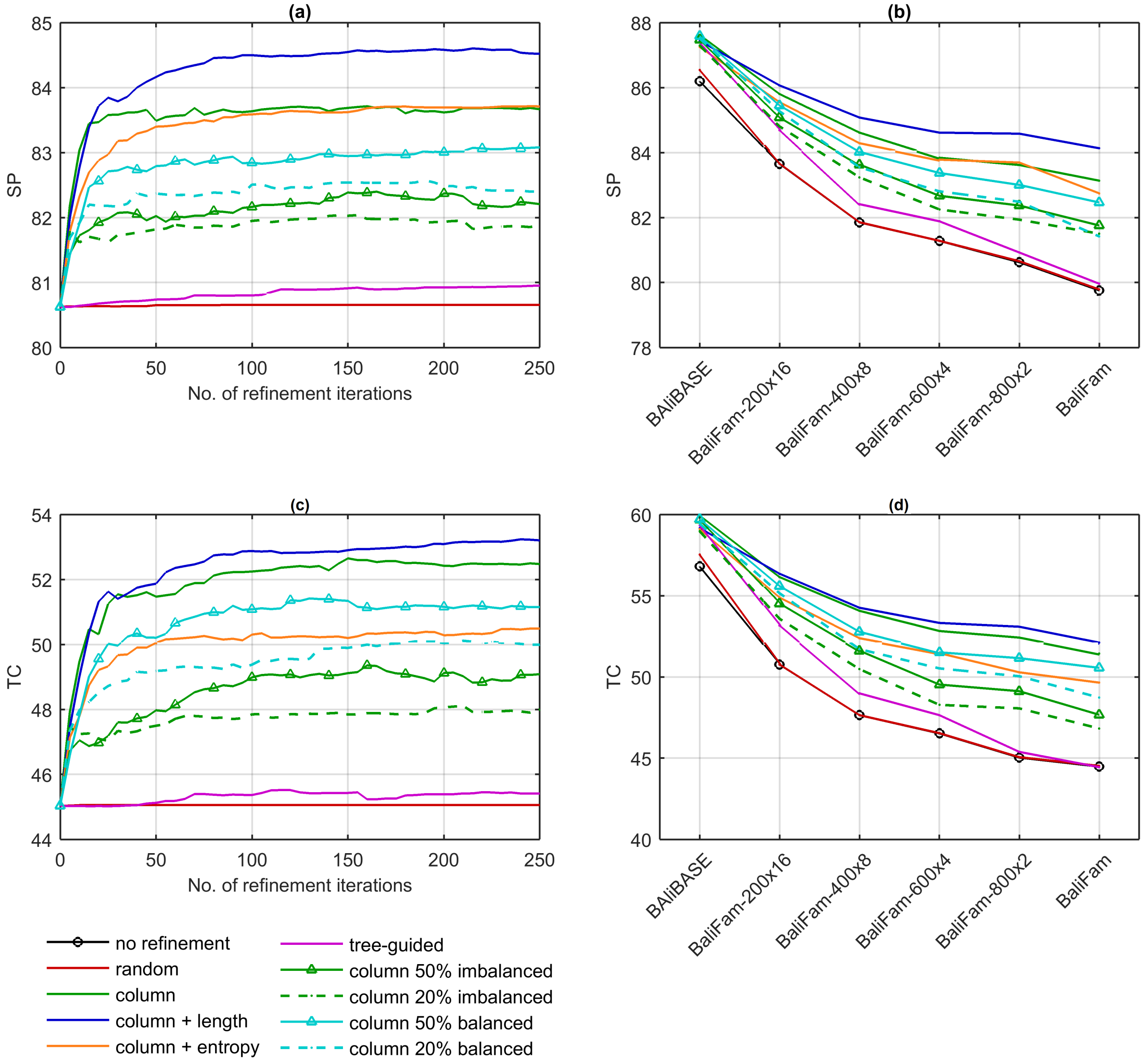

Supplementary Figure 3: Comparison of different refinement strategies: effect of consecutive iterations on BaliFam-800x2 (a, c), scalability with respect to the number of sequences after 200 refinements (b, d).

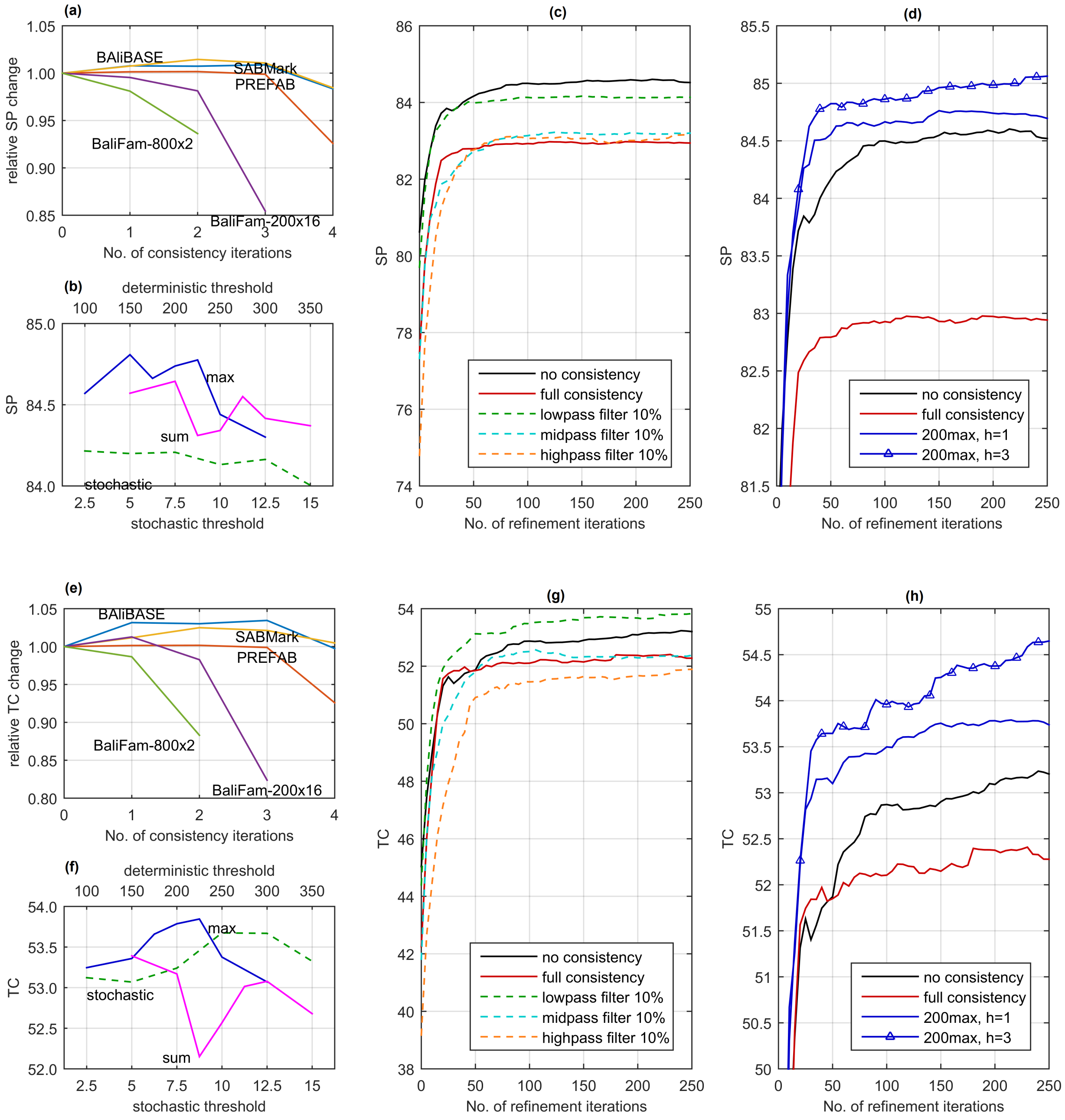

Supplementary Figure 4: Effect of consistency iterations on selected benchmarks (a, e). Analysis of consistency on BaliFam-800x2: effect of distance-related filtering (c, g), selectivity variants for closely related sequences (b, f), weighting original posterior matrices by $h_{xy} \in \langle 1, h \rangle$ coefficient (d, h). Qualities from charts (a), (e), (b), and (f) measured after 200 refinements.

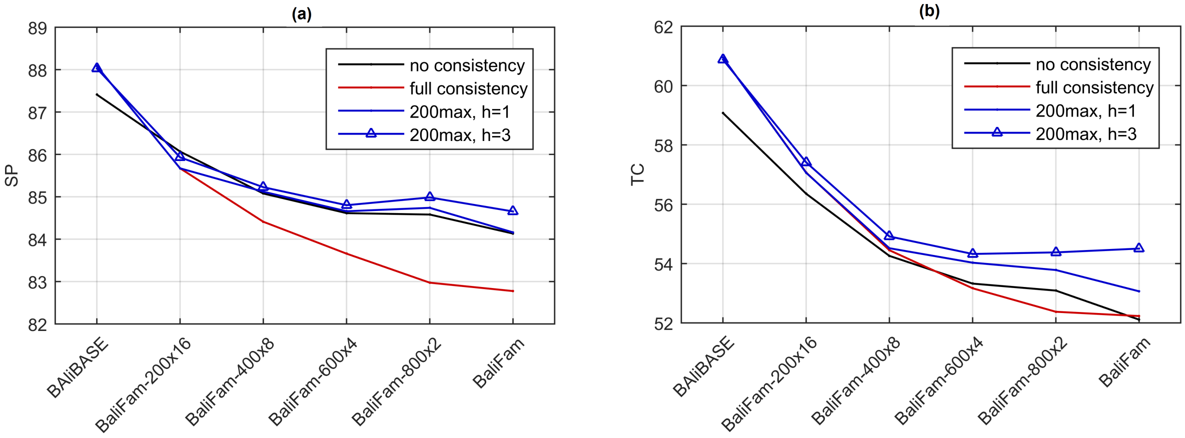

Supplementary Figure 5: Scalability of consistency: sum of pairs (a) and total column (b) scores after 200 refinements.
